# Urinary Stent Development and Evaluation Models: In Vitro, Ex Vivo and In Vivo—A European Network of Multidisciplinary Research to Improve Urinary Stents (ENIUS) Initiative

**DOI:** 10.3390/polym14091641

**Published:** 2022-04-19

**Authors:** Noor Buchholz, Alberto Budia, Julia de la Cruz, Wolfgang Kram, Owen Humphreys, Meital Reches, Raquel Valero Boix, Federico Soria

**Affiliations:** 1 U-Merge Scientific Office, U-Merge Ltd. (London-Athens-Dubai), Menandrou Street, 14561 Athens, Greece; 2 Department of Urology, “La Fe” Polytechnic University Hospital, Avinguda de Fernando Abril Martorell, 106, 46026 Valencia, Spain; alberto.budia@hotmail.com; 3 Jesús Usón Minimally Invasive Surgery Centre, Carretera N-521, km. 41.8, 10071 Cáceres, Spain; jecruz@ccmijesususon.com (J.d.l.C.); raquelvb4@gmail.com (R.V.B.); fsoria@ccmijesususon.com (F.S.); 4 Department of Urology, University Medical Center Rostock, Ernst-Heydemann-Straße 6, 18057 Rostock, Germany; wolfgang.kram@med.uni-rostock.de (W.K.); 5 UCD Centre for Biomedical Engineering, University College Dublin, Stillorgan Rd., Belfield, D04 V1W8 Dublin, Ireland; owen.humphreys@ucdconnect.ie; 6 Institute of Chemistry, The Center for Nanoscience and Nanotechnology, The Hebrew University of Jerusalem, Jerusalem 9190401, Israel; meital.reches@mail.huji.ac.il

**Keywords:** urinary stent, design, material, encrustation, in vitro, ex vivo, in vivo, evaluation, animal models, urinary tract models

## Abstract

*Background*: When trying to modify urinary stents, certain pre-clinical steps have to be followed before clinical evaluation in humans. Usually, the process starts as an in silico assessment. The urinary tract is a highly complex, dynamic and variable environment, which makes a computer simulation closely reflecting physiological conditions extremely challenging. Therefore, the pre-clinical evaluation needs to go through further steps of in vitro, ex vivo and in vivo assessments. *Methods and materials*: Within the European Network of Multidisciplinary Research to Improve Urinary Stents (ENIUS), the authors summarized and evaluated stent assessment models in silico, in vitro, ex vivo and in vivo. The topic and relevant sub-topics were researched in a systematic literature search in Embase, Scope, Web of Science and PubMed. Clinicaltrials.gov was consulted for ongoing trials. Articles were selected systematically according to guidelines with non-relevant, non-complete, and non-English or Spanish language articles excluded. *Results*: In the first part of this paper, we critically evaluate in vitro stent assessment models used over the last five decades, outlining briefly their strengths and weaknesses. In the second part, we provide a step-by-step guide on what to consider when setting up an ex vivo model for stent evaluation on the example of a biodegradable stent. Lastly, the third part lists and discusses the pros and cons of available animal models for urinary stent evaluation, this being the final step before human trials. *Conclusions*: We hope that this overview can provide a practical guide and a critical discussion of the experimental pre-clinical evaluation steps needed, which will help interested readers in choosing the right methodology from the start of a stent evaluation process once an in silico assessment has been completed. Only a transparent multidisciplinary approach using the correct methodology will lead to a successful clinical implementation of any new or modified stent.

## 1. Introduction

The human urinary system consists of four main components: the kidneys, ureters, urinary bladder and the urethra. The kidneys filter the blood plasma and produce urine. The urine is drained to the bladder through the ureters where it is stored until its excretion from the body through the urethra. The ureters are muscular tubes, approximately 250–300 mm long that transport the urine produced by the kidneys to the bladder. Their diameter can vary from 1 to 10 mm along their course between the renal pelvis and the bladder [1]. The proximal end of the ureters (the pelvis) is located in the renal hilum and splits into the major and minor calyces [2]. As the renal calyces and the proximal regions of the ureters become distended due to the accumulation of urine, a peristaltic contraction is initiated. This begins in the calyces and spreads down to the renal pelvis and along the length of the ureter and pushes the urine down the ureters into the bladder [3]. The frequency of the peristaltic contractions, and thus the rate of urine flow through the ureters depends on the rate of urine filtration by the kidneys and the bladder pressure [4].

Urological stents are hollow tubes that are inserted into the ureters to maintain urinary drainage from the kidney and overcome obstructions [5]. They are used extensively in urology to provide a minimally invasive treatment for a wide range of indications including kidney stones, tumors, strictures and infection. Stents can also facilitate healing as a scaffold or be used as a prophylactic measure against stricture formation [6]. There is a wide range of urinary stent designs, materials, and coatings with implications on the physico-chemical properties of each stent. A number of complications are associated with urinary stents including pain, infection and migration. The “ideal” stent should be stable following placement, radio-opaque, resistant to infection and encrustation, and provide long term drainage [5].

Ureteral stents have been used in urology for more than 40 years and are most commonly used for the prevention and treatment of obstruction caused by kidney stones. The stent is either inserted before or after the surgical removal of a stone by ureterorenoscopy, percutaneous nephrolithotomy or shockwave lithotripsy (SWL) [7].

Prophylactic stenting allows larger stone particles to pass through the urinary tract without causing obstruction and reducing infection [5]. Ureteral stents can promote healing in the ureters, by realignment of the ureter walls and acting as a scaffold for epithelial growth [8]. Insertion of the stents causes relaxation of the ureter and thus increases the passage of urine further [7].

Most ureteral stents have coiled ends to prevent migration. The coiled ends have shape memory properties and regain their shape after insertion. The most common designs are the pigtail, J, cross-coil and double pigtail. The first double-ended pigtail stents were introduced in 1978. This design still is the gold standard, and the double-J is the most widely used ureteral stent [6]. These stents provide efficient drainage along the full length of the ureter. Most stents are made of polyurethane or silicone. They have a high tensile strength, good biocompatibility and a low coefficient of friction. They also feature multiple side holes for improved drainage and flow. The incorporation of hydrophilic coatings provides a smoother surface, reduces friction and minimizes encrustation.

Urological stents have become an indispensable tool in the armamentarium of urologists. However, especially when considering stents for a longer use, problems remain with infections, biofilm formation and encrustation, urothelial reaction in the form of hyperplasia, migration and patient intolerance. Continuous efforts in the research and development of these stents try to alleviate some of the problems by modifications of stent designs, materials, coatings and lengths, but so far only with limited success in clinical practice [9].

When trying to invent, develop or improve urinary stents, certain steps have to be followed before clinical evaluation in humans. Usually, the process starts with computer modeling and in silico simulation assessment. However, the urinary tract is a highly complex, dynamic and variable environment. Factors such as constant urodynamic and physico-chemical changes, peristalsis, stent–related vesico-ureteric reflux, and the multifactorial nature of urinary infections, biofilm formation and encrustation make a computer simulation extremely challenging [10,11,12,13].

Therefore, each pre-clinical evaluation needs to go through the further steps of in vitro, ex vivo and in vivo assessments.

In vitro stent assessment models usually look at the encrustation and biofilm formation on urinary stents either in human or in artificial urine. However, inherently, they are very limited in accurately reflecting (patho-)physiological conditions. Many models have been developed and tried over the last few decades, each having certain strengths and limitations. In this paper, we attempt a critical evaluation of these models.

After in vitro evaluation, the next step should be ex vivo models where stents are tested in, partially, functional organs. We attempted to summarize the necessary steps to set up such a model using as an example a biodegradable stent.

After completion of these two steps, the final assessment before human trials should be animal models. There are many models in various animal species available, and we attempt to discuss the advantages and disadvantages of these models for the testing of urinary stents.

The aim of this paper is not to present yet another review on stent evaluation models. These are already available in the literature. Rather, we aim to provide a practical overview and critical discussion of the experimental pre-clinical evaluation steps needed. We hope that will help interested readers to choose the right methodology from the start of a stent evaluation process once an in silico assessment has been completed.

## 2. Materials and Methods

The European Network of Multidisciplinary Research to Improve Urinary Stents (ENIUS) is a collaborative project supported by the European Collaboration in Science and Technology (COST). This is a four-year project enabling direct collaboration between clinicians and scientists from multiple disciplines and from most European and many neighboring countries. Within ENIUS, working groups were formed to evaluate the currently available stents, their clinical application, in silico simulation of fluid dynamics, stent materials and coatings, drug-eluting technology, and future research approaches. The authors of this paper attempted to evaluate three steps of pre-clinical stent evaluation: namely in vitro, ex vivo and in vivo testing.

Sub-topics were researched in a systematic literature search in the databases of Embase, Scope, Web of Science and PubMed. Clinicaltrials.gov was consulted for ongoing trials.

**Figure A1:**
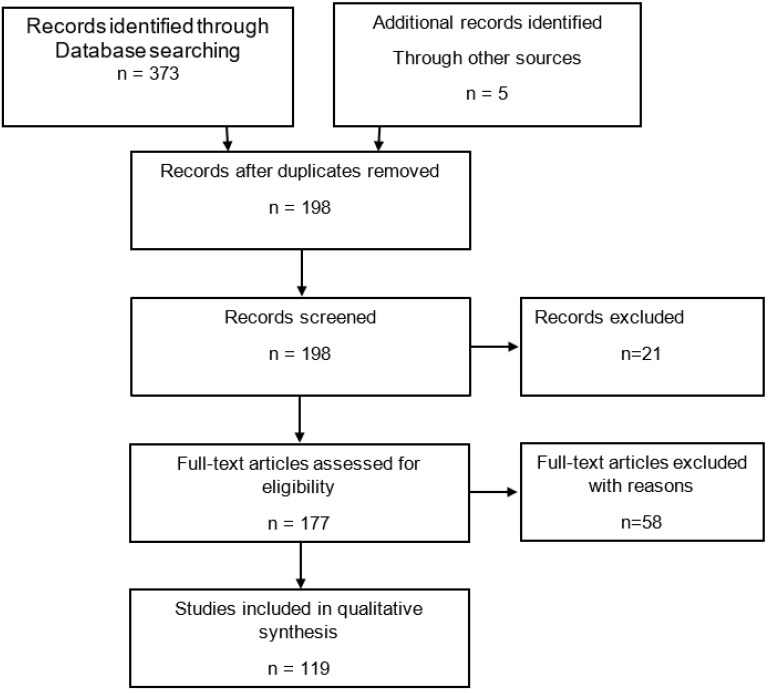
PRISMA flow algorithm; search of databases: PubMed, Scopus, Web of Science. Keywords: in vitro, encrustation, artificial urine, ureteral, urethral, stent, catheter, in combination with Boolean operators; time period until 2021. Exclusion criteria: non-relevant, non-English articles, no abstract available, non-full-text.

For in vitro models, search terms were “in vitro, encrustation, artificial urine, ureteral, urethral, stent, catheter, in combination with Boolean operators” until 2021. From the initial 373 articles found, 119 were finally used for this review after excluding duplicates, non-relevant, non-English, lacking abstracts or non-full text publications (Figure A1).

**Figure A2:**
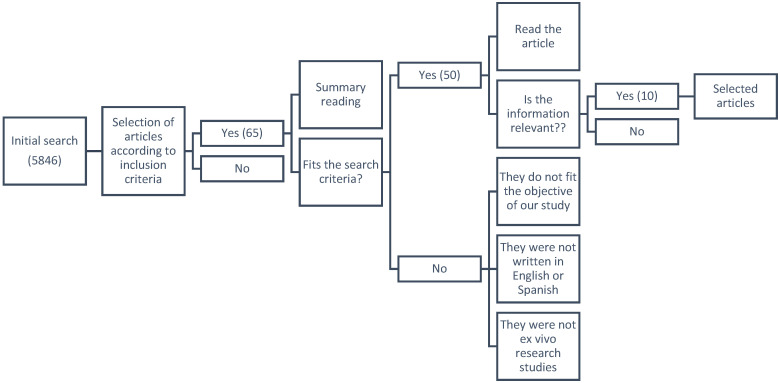
PRISMA flow algorithm; search of databases: PubMed, Scopus, Web of Science. Keywords: stent, design, material, encrustation, biodegradable, ex vivo, ureter, urinary tract models; time period until 2021. Exclusion criteria: non relevant or not meeting objective of our study or not ex vivo, non-English or non-Spanish articles, no abstract available, non-full-text.

For ex vivo models, search keywords were “stent, design, material, encrustation, biodegradable, ex vivo, ureter, urinary tract models” until 2021. The initial search produced 5846 articles. Abstracts and if necessary full texts were reviewed, 5836 articles were hence excluded on the basis that they were not relevant, did not meet study criteria, were not ex vivo studies, were not written in English or Spanish, or either the abstract or the full text were not available. Three systematic reviews, one clinical study and six articles were selected for inclusion (Figure A2).

**Figure A3:**
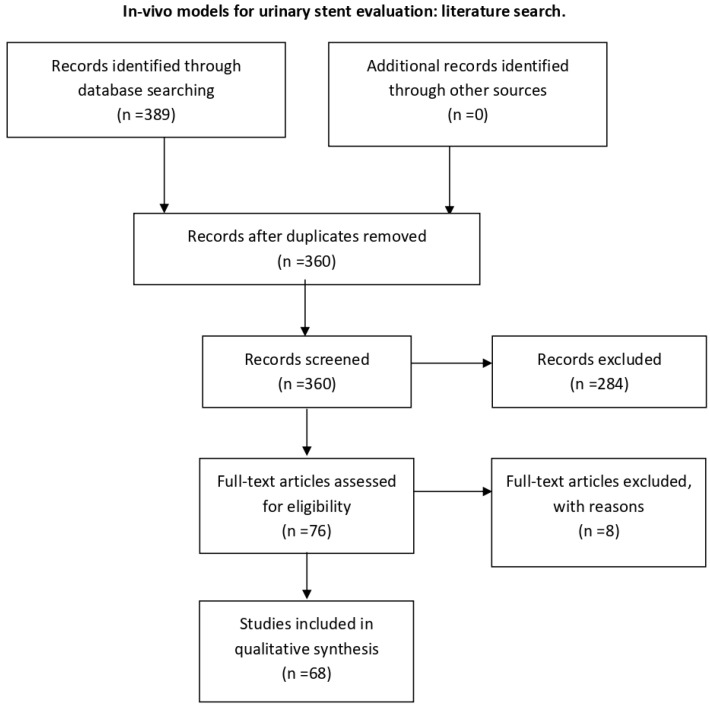
PRISMA flow algorithm; search of databases: PubMed, Scopus, Web of Science. Keywords: validation; testing; urinary stent; ureteral stent; urethral stent; animal model; in vivo; time period until 2021. Exclusion criteria: non-relevant, not in vivo, non-English, no abstract available, non-full-text.

For in vivo models, the following search terms were used: “validation OR testing” AND “urinary stent OR ureteral stent OR urethral stent” AND “animal model OR in vivo”. The initial search retrieved 389 articles, of which 68 were included for this review after screening and full-text eligibility assessment and removal of duplicates (Figure A3).

## 3. In Vitro Stent Encrustation Models

### 3.1. Background

Implantation of biomaterials into the urinary tract is hampered by crystal formation, bacterial adhesion and, ultimately, encrustation through biofilm formation resulting from a multifactorial disturbance of the delicate balance between numerous physico-chemical and biochemical processes. Non-infectious stone formation and encrustation usually result from metabolic imbalances often on the tubular level. In contrast, infectious stone formation and biofilm-induced encrustation are linked to the enzymatic activity of bacteria. Best known are urease-producing species such as *proteus mirabilis*, which increase the pH of the urine. This alkalization, in turn, decreases the solubility of urinary calcium and magnesium salts and thus facilitates encrustation. Consequently, the use of urinary implants is complicated by several factors such as stent surface encrustation through the deposition of crystal-forming urinary ions, bacterial colonization and biofilm formation despite antibiotic treatment and prophylaxis, mechanical irritation of the urothelium and alterations of urine flow in and around the stent.

### 3.2. Past and Current In Vitro Systems and Their Limitations

The development of in vitro models to simulate bacterial infections and biofilm formation started after the initial observation of sessile bacteria and their role in chronic infections in humans. Biofilms form an irregular network that allows the exchange of nutrients and genetic materials among bacteria. They protect bacteria from physical, chemical and biological stresses. Shear stress caused by the flow of the fluid in the biological medium is hereby one of the main factors impacting on the formation of a stable biofilm.

Early approaches focused on the use of continuous flow systems, such as the Chemostat model, which had the advantage of a regular supply of fresh fluid medium whilst maintaining a constant volume [14]. Many in vitro models designed to mimic encrustation on urological devices have been derived from classical microbiological approaches and often do not reflect important physiological factors such as the complex and variable physico-chemical urinary environment in vivo, or infection with mixed species.

In 1973, Finlayson and Dubois described a dynamic flow in vitro encrustation model which used both a constant flow of artificial urine and a magnetic stirrer [15]. Several adaptations to this model have been devised over time to enable the study of urinary encrustations utilizing both human and artificial urine [16].

Depending on the particular research question, two groups of open systems (fed batch process) were designed: the Plug Flow Reactor (PFR) and the Continuous Flow Stirred Tank Reactor (CFSTR). The PFR enables the characterization of constant-flow systems. Fluid moves as a row of coherent “plugs” in an axial direction inside the reactor. Each “plug” can have a different composition [17].

The Modified Robbins Device (MRD) is such a PFR system and was designed to monitor biofilm formation with different flow speeds in an axial direction and in a completely mixed reactor using diffusion. This system consists of a pipe with multiple threaded holes containing coupons [18]. Gorman et al. described a dynamic constant flow encrustation model using artificial urine and a MDR [19]. When used with an upstream Chemostat, this system is the current gold standard to evaluate surface-modified stents and catheters under dynamic experimental conditions [20].

The biofilm reactor of the Center for Disease Control (CDC) is an up-to-date commercially available flow-based CFSTR-system. A vessel with a polyethylene lid bears independent rods housing removable coupons. Inside the reactor, there is a rotating magnetic stirrer exerting a constant high shear force on the coupons similar to intraluminal urine flow in a catheter. The system allows for perfect mixing and operates at a steady state [21]. The structure and physiology of biofilm formation can be monitored by confocal laser scanning microscopy (CLSM) in a non-invasive fashion.

MRD and CDC biofilm reactors are indispensable for prototype testing but less suitable for screening testing. A disadvantage of these semi-open designs is their susceptibility to contamination. This led to the development of high-throughput static biofilm models. Microtiter plate (MTP)-based static systems are perhaps the most commonly used biofilm model systems. They are an important tool to study especially the early stages of biofilm formation. They are particularly useful to examine the early stages of biofilm formation. In these systems, biofilms are typically grown on either the bottom or the side walls of a MTP. MTP-based systems are closed systems without in- or outflow (batch process). Consequently, during an experiment, the composition of the environment inside the well of an MTP changes. Nutrients are depleted whilst signaling molecules accumulate. It has been suggested that a part of the accumulated biomass may not result from biofilm formation but rather from cell sedimentation and the subsequent entrapment of cell sediments within the matrix of extracellular polymeric substances (EPS) [22,23,24].

The Calgary Biofilm Device (CBD) represents a modification of the MTP-based systems, where biofilms are formed on lids with rods that fit into the bacteria-containing wells of the MTP [25]. A newer system uses this CBD as a commercially available high-throughput screening assay to study biofilm formation and encrustation on implants. However, the lid is configured in such a way that materials are held in a matrix. The bottom is a well plate into which the implant materials to be tested are inserted. The matrix in combination with the high-throughput capability of the assay allows the study of several encrustation parameters [26]. The use of MTP-based assays offers many advantages. MTPs are inexpensive and they provide the opportunity for multiplexing, as multiple organisms and treatments can be incorporated in a single experimental run [23].

Both MTP/CBD-based and flow-based systems share some limitations. One common pitfall in designing in vitro biofilm models is the use of bacterial strains (usually only one) with a low virulence which, in turn, results in a low translation rate from in vitro to in vivo studies.

Most in vitro encrustation models use synthetic urine based on urease reactions or urease-producing bacteria. However, in real life, most urinary tract infections are caused by *E. coli*. These are acid-producing and, consequently, do not increase the urinary pH. While models using urease-related alkalization are relatively easy to design, the multifactorial physiological conditions in stone and encrustation formation are not properly represented. In fact, 80% of all urinary stones and probably most urinary implant encrustations mainly consist of calcium and oxalate. Only 10% of urinary stones contain uric acid crystals, and struvite as a typical infectious stone is clinically found in less than 10% of urinary stones, typically in alkaline urine with a pH > 7 [27]. Yet, alkalization models do focus on this group of stones.

In clinical practice, guidelines mandate that urinary catheters and stents with infectious stone encrustations must always be removed due to the presence of inactive bacteria protected by the biofilm [28].

The above-mentioned encrustation models could be complemented by in vitro calcium oxalate crystallization methods from urolithiasis research. There are different options to choose from. These vary from simple experiments in defined inorganic solutions to whole human urine experiments replicating urine flow dynamics [29,30,31].

**Figure 1:**
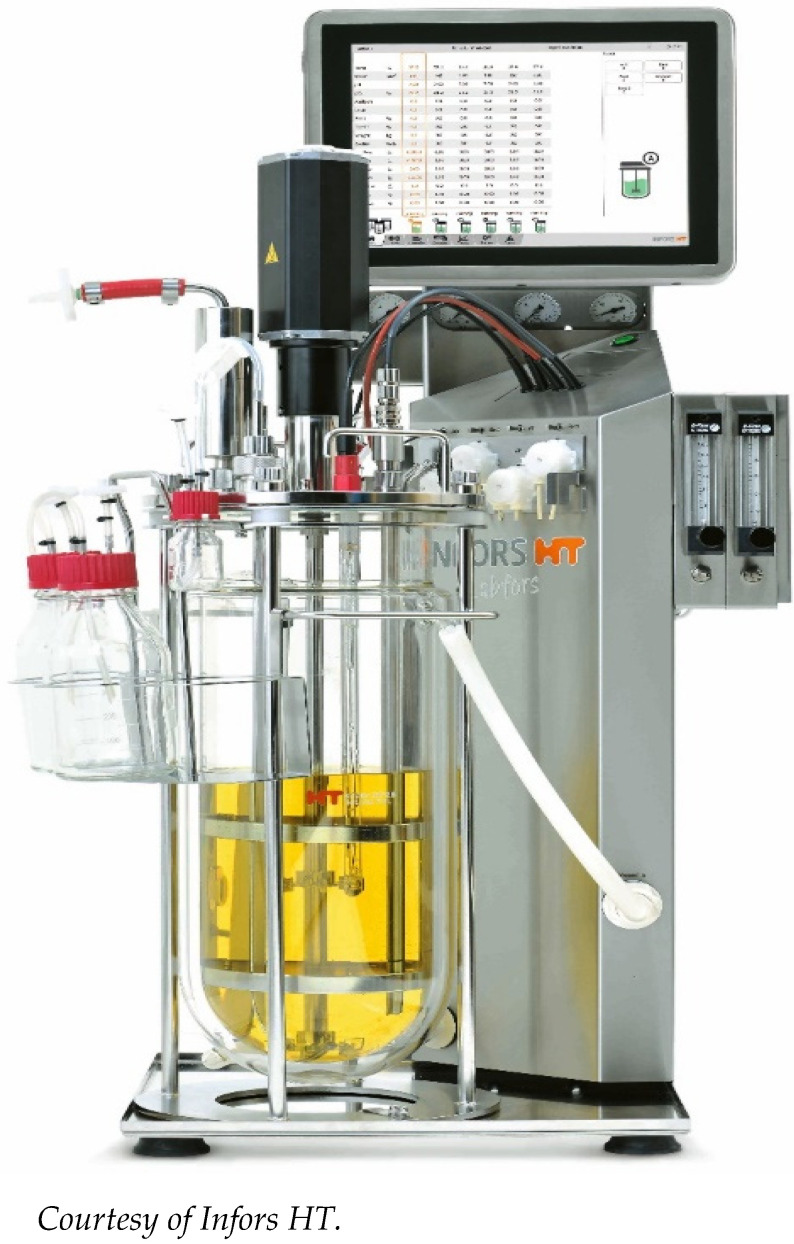
Bioreactor Labfors 5.

**Figure 2:**
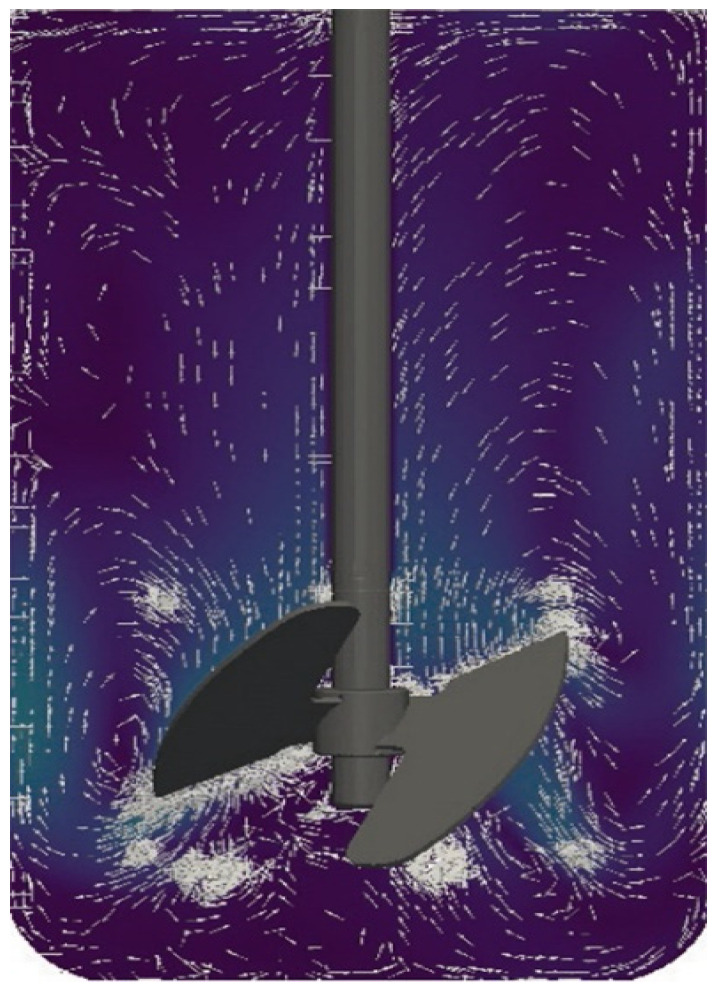
Flow modeling.

Currently, models are being developed that combine the advantages of both continuous flow and static models. One such system is the Stent-on-Chip microfluidic model (SOC). SOC tries to simulate the hydrodynamic areas of a stented ureter under physiological conditions including drainage holes and the cavity formed by a ureteral obstruction. In addition, Computational Fluid Dynamic (CFD) simulations helped to determine the spatial distribution of the flow fields and the deposition of encrustation within the SOC architecture. The encrustation over time is monitored and quantified by means of optical microscopy [32]. Currently established qualifying and quantifying methods to identify and analyze bacterial adhesions, biofilm formation and encrustations have been evaluated elsewhere [23,33]. CFD simulations open new perspectives also for CFSTR. Using an adapted version of the bioreactor Labfors 5 (Infors AG, Bottmingen, Switzerland) (Figure 1), fluid currents can be simulated mimicking physiological ureter reactions after the insertion of a stent (Figure 2) [34].

To date, in vitro encrustation models are still the best option for the evaluation of new biocompatible materials to assess their resistance to infection and encrustation. Table 1 lists selected models and their advantages and limitations [35,36,37,38,39,40,41,42,43,44,45,46,47,48,49,50,51,52,53,54].

## 4. Ex Vivo Stent Evaluation

### 4.1. Background

One of the most common ideas in the field of urinary stents is to equip them with biodegradable properties to reduce the probability of stent failure and associated problems such as forgotten stents, encrustation and stent-related morbidity. New prototypes, coatings, and biomaterials are constantly developed to improve the biocompatibility of these internal devices. The combination of precise knowledge of physiopathological mechanisms and bioengineering to allow the creation of drug-eluting biocoated stents or new biodegradable materials that reduce or diminish undesired stent effects may be a good way to create a near-to-ideal stent [55,56].

Although in silico experiments are useful to facilitate the teaching of fundamentals, they cannot accurately reflect a clinical situation. The use of biological models is a more appropriate approach before testing in humans. The main aim of this section is to understand some important concepts of ex vivo methodology in clinical evaluation and bioengineering, thereby encouraging multidisciplinary collaboration to provide complementary tools to assist in the development of new urinary stents.

### 4.2. Steps to Successful Ex Vivo Research on Urinary Stents

#### 4.2.1. Material-Related Aspects

Design requirements define target values for properties that constrain the design, such as physical, mechanical, functional, chemical, and eco-properties. Therefore, we will choose a material according to its characteristics that meet these objectives and which is compatible with the experimental set-up and process.

In medicine, two types of materials are used, namely natural and synthetic polymers. Depending on their necessary indwelling and functionality time, materials are either biostable or biodegradable. The former is used when these times are indefinite; the latter is used when these times are limited [10,57].

For the design of a biodegradable urological prosthesis, we must take into account that:

- The material must not be absorbed;
- Dissolution must be complete without obstructive non-dilutable fragments;
- The device must be rigid enough to facilitate its placement;
- It must be radiopaque and echogenic to facilitate follow-up imaging;
- It may incorporate a urine color marker that indicates the degradation rate (ideally not reddish in color).

In addition, the polymer used must be characterized by:

- A controlled rate of degradation;
- Adequate biomechanical resistance to the urinary tract wall;
- Degradation by-products being non-mutagenic, -antigenic, and -toxic;
- Sterilizability;
- Biocompatibility (i.e., not generating an immune response from the host).

Once the stent material has been chosen according to these criteria, it must first be tested in the laboratory. The most commonly used methods for the characterization of materials prior to biological testing are described below [58,59].

##### Materials Characterization for the Evaluation of Antimicrobial or Antifouling Activity

- Surface chemistry

Preconditioning and characterization of the material by simulating different body fluids is performed using standardized solutions of clinical relevance, which is followed by analytical methods for chemical characterization of the surface after such pre-treatment. Methods include among others attenuated total reflectance Fourier-transform infrared spectroscopy (ATR-FTIR), grazing-angle FTIR, atomic force microscopy (AFM) and X-ray photoelectron spectroscopy (XPS).

- Surface physico-chemical properties

To assess the physico-chemical properties of a material surface, parameters such as surface tension, hydrophobicity, surface charge, morphology, topography and surface roughness are measured. These are very important for the adhesion of microorganisms to the material.

- Physico-mechanical properties

To assess physico-mechanical properties, the elasticity, opacity and hardness of the material must be analyzed. For drug-eluting materials, the pharmaceutical characterization with the evaluation of the amount of active compound released over time needs to be specified.

##### Biocompatibility and Cytotoxicity Testing

For cytotoxicity and biocompatibility assays, the guideline is to follow ISO-10993 standard recommendations.

### 4.3. Microbiological Testing In Vitro

There are several methods for testing and validation of antimicrobial designs. Some are standardized; others are specific tests. Certain criteria apply:

- The incubation medium must mimic the conditions in the host.
- The device must be in a near-clinical situation (i.e., profiles of microorganisms representative of the urinary tract must be used, and the device must be under clinical flow conditions).

Regardless of the microbial test used, there must always be an evaluation after bacterial or fungal exposure. To assess the metabolic activity, one may use cell viability assays such as MTT (3-(4,5-dimethylthiazol-2,5-diphenyl-2H-tetrazolium bromide) or XTT (cell proliferation kit II) assays. For bacterial cell viability, a colony-forming unit (CFU) count is used, and microscopy identifies the substance and lists the characterization and morphology.

### 4.4. Material Release

In the case of evaluating a biodegradable material, the quantification of the release of this product must be added to the test. To quantify the amount of polymer released, one method of measurement frequently used is to incubate sample discs individually in a 48-well plate with 600 μL of Milli-Q (MQ) water at 37 °C. Every 24 h, 600 μL of MQ water is collected for measurement and replaced over a period of 5 days successively until day 10. Polymer measurements are made with absorbance measurements according to the material used.

### 4.5. Computational Modeling

**Figure 3:**
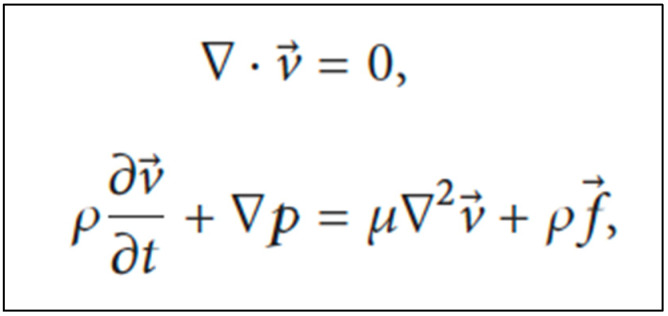
Navier–Stokes equations to investigate the movement of urine within the ureter. Legend: v = spatial velocity vector; *ρ* = fluid density; *p* = static pressure; *μ* = dynamic viscosity; *f* = body force vector.

For the investigation of the movement of urine flow within the ureter, many methods have been studied. The finite element method and fluid–structure interactions are normally used. However, we suggest the CFD method to help during this design process, as it can simulate the behavior of different biological tissues and provide a realistic computational environment for evaluating stents, taking into account the length and curvature of a ureter from the ureteropelvic junction (UPJ). Assuming that peristalsis is suppressed in the stented ureter and, therefore, active wall contractions are ignored, and the ureter wall is modeled as an elastic membrane with a natural cross-sectional area, urine is considered to be a Newtonian and incompressible fluid with laminar flow. The Navier–Stokes equations are used to investigate the movement of urine within the ureter (Figure 3) [10,11,12,60,61]

### 4.6. Aspects Related to Size and Diameter

Determining the appropriate ureteral stent length is very important to reduce stent-related complications. This parameter can be measured by radiography where we measure the distances of the kidney, ureter and bladder. For patients with a ureter length of ≥29.4 cm, a 26 cm ureteral stent has been found appropriate. For ureters 27.1 to <29.4 cm, a 24 cm, and for ureters <27.1 cm, a 22 cm ureteral stent is indicated, respectively.

As for stent diameter, a study of the catheter flow rate in 5, 6, 7 and 8 Fr double-J stents found that the total flow rate in 5 Fr double-J stented ureters was higher than in ureters with double-J stents of other sizes [62].

## 5. In Vivo Models for Stent Evaluation

### 5.1. Background

Experimental in vivo trials represent the final step in the pre-clinical validation of medical devices. These in vivo evaluations should be preceded by the corresponding in silico simulations, in vitro, and ex vivo studies of the newly developed device. The urinary tract constitutes a complex dynamic environment with high variability, where in vitro and ex vivo models often fail to reflect certain factors that are decisive for the safety and effectiveness of the urinary stent. These factors include urodynamic behavior of the urinary tract, the ever changing physico-chemical conditions, and the multifactorial nature of urinary tract infections, biofilm formation and encrustations. In addition, ureteral peristalsis and the potential presence of vesicoureteral reflux may play a crucial role in the success of new designs of ureteral stents [63,64,65].

Prior to its translation into a clinical setting, the safety and performance of a urinary stent are required to be tested in a whole organism, which is provided currently by animal models. Animal models overcome the aforementioned limitations of reproducibility in a laboratory setting and also allow the evaluation of the systemic effect of a new device on the host, including its potential systemic toxicity [66].

The rational sequence of pre-clinical assessment of a new stent design or innovation should follow the steps from in silico, in vitro and ex vivo studies, to finally in vivo trials. This thus allows the reduction in the number of animal models necessary to a minimum, still providing adequate statistical power, increasing the likelihood of success of these experimental trials, and at the same time preserving animal welfare [67,68].

Concerning animal welfare in experimental studies, the ethical evaluation of projects involving animal testing is mandatory in the EU since January 2013, through the Directive 2010/63/EU of the European Parliament and of the Council establishing the basic rules applicable to the protection of animals used in experimentation and other scientific purposes [68,69]. To ensure moral standards, scientific validity, and public trust, all projects must be evaluated and approved by an ethical committee. The use of animals for research should be justified by carefully evaluating each procedure as to its scientific validity, usefulness and relevance of the expected result. The potential harm to the animal will be balanced against the expected benefits of the project [68,70].

The specific aims of this chapter were: (1) to report which species of laboratory animals are being used for the validation of urinary stents; (2) to describe what features of these species provide reliable conditions for this testing; (3) to enumerate the type of stent validated in each of the laboratory animal described; (4) to evaluate the existence of models that simulate certain conditions such as urological lesions or processes; and (5) to describe the aspects assessed in the studies, as well as the diagnostic tests these animals undergo.

### 5.2. Currently Available In Vivo Models—Applications and Limitations

Regarding the translational perspective of animal research, the choice of species should be based on the similarity of the medical conditions studied with those in the human body. Ideally, we should look for a model that provides anatomic, urodynamic, pathophysiological, histological and biochemical levels as identical as possible to that of humans.

#### 5.2.1. Non-Human Primates

Non-human primates represent the closest model in this regard, except for two anatomic variations: they possess unipapillary kidneys, and the left kidney lies lower in the abdomen, unlike human kidneys [71]. Nevertheless, the scientific literature has not reported the assessment of urinary stents in primates, which may be due to ethical, legal, economic and logistical considerations [69,72].

#### 5.2.2. Pigs

The porcine species are the most frequently used animal model for the assessment of urinary stent designs. The anatomy of the human and porcine urinary tracts is highly similar, rendering this model ideal for analyzing the behavior of the urinary tract in the presence of new devices [73]. Pigs have multipapillary kidneys, with 8–12 papillae, while humans usually have 4–18 [74]. Porcine ureters tend to be longer and more tortuous than those of humans [73,75,76]. Moreover, porcine renal physiology parallels that of humans with respect to maximal urine concentration, glomerular filtration rate and total renal blood flow [77]. Since the male porcine urethra prevents a retrograde approach due to its sigmoid morphology, research involving endourologic procedures is performed on female pigs. Ideally, interventions should be carried out on 35–40 kg models, as the dimensions of their urinary tract at that weight are comparable to a human adult [78,79].

**Figure 4:**
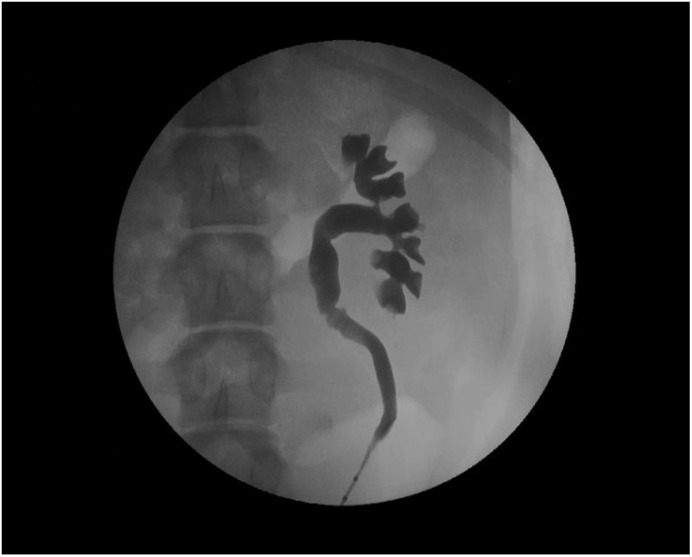
Retrograde ureteropyelography of a porcine left nephroureteral unit.

The devices assessed in the porcine model are mainly ureteral stents such as polymeric, antireflux, biodegradable, drug-eluting, and metallic stents [77,78,79,80,81,82]. Usually, stents are inserted retrogradely, although antegrade and cystostomy approaches have been described [77,81,82,83,84,85]. The evaluation of the stent performance in vivo involves blood and urine analysis, urine culture, and imaging tests that include the ultrasonographic assessment of the degree of hydronephrosis [86]. Other radiologic tests such as excretory urography and retrograde ureteropyelography can provide valuable information on urinary patency, stent migration, radiopacity and mode of degradation of biodegradable devices (Figure 4) [65,86,87].

On the other hand, the pig model cannot assess vesicoureteral reflux by voiding cystourethrography, which can however be examined via a simulated voiding cystourethrography [64,88].

In the pig model, histology can assess biocompatibility, tissue damage and, more specifically, ureteral healing with a stent in situ [87,88,89]. In addition, intravesical and intrarenal pressures with a ureter stent can be measured as well as changes in ureteral peristalsis and contractility [82,90,91]. Urinary stent research in the porcine species is generally performed on healthy intact models. However, pigs may undergo the surgical and pharmacological induction of pathologic features such as ureteral strictures and urolithiasis [84,88,92]. Moreover, recent studies have also chosen the porcine species for the assessment of instillation methods for topical therapies to the upper urinary tract as an adjuvant treatment for non-muscle invasive upper urinary tract urothelial carcinoma, which include ureteral stents [93,94].

In contrast, the validation of urethral and prostatic stents is generally not performed in pigs given the particularities of the male porcine urethra and the anatomical differences of the accessory sex glands [75].

#### 5.2.3. Dogs

The dog has proven to be an adequate model for the study of prostate diseases, as it develops benign prostatic hyperplasia (BPH) and prostate cancer both spontaneously and experimentally induced [95,96]. Metallic, covered, drug-eluting and biodegradable urethral stents have been assessed in healthy and in BPH-induced canine models via transurethral insertion [97,98,99,100]. The urethral diameter is measured utilizing a retrograde urethrography which enables the monitoring of position, expansion, patency and migration of the stents [98,99,101]. Histology may assess stent-related urethral damage and urothelial hyperplasia [98,102]. The use of urodynamic studies for testing the therapeutic response in BPH canine models is however not reliable since, contrary to the human prostate, canine BPH produces rectal obstruction rather than lower urinary tract symptoms [96].

The canine model has occasionally been chosen for the evaluation of biodegradable ureteral stents. Lumiaho et al. tested their first prototypes of their biodegradable ureteral stent in dogs, inserting the stents with an open surgical approach. In addition to radioisotope renal scans, analysis of renal function, ureteral patency and the presence of vesicoureteral reflux were carried out similarly to the methodology described in pigs [103,104,105].

#### 5.2.4. Smaller Animals

Smaller laboratory animals, such as rabbits and rats, provide the advantages of easier handling, are more cost-effective and require less infrastructure and logistics [92]. Unlike porcine and canine models, whose dimensions and anatomy allow the evaluation of the urinary stents that may be tested in future clinical human trials without modifications, the devices inserted in rabbits and rats may differ in form and size from the definitive prototype under development. Small laboratory animals are therefore of great use for the assessment of stent upgrades such as biomaterials, coatings and the release of substances [13,106,107].

##### Rats

As for the rat model, it enables the analysis of the antimicrobial and anti-encrustation potential of new stents, since urolithiasis and urinary tract infection (UTI) can be experimentally induced in a controlled manner [13,96,105]. UTI models are induced by the intravesical instillation of bacterial suspensions, most commonly *S. aureus*, *E. faecalis* and *P. aeruginosa* [107,108,109]. The induction of urolithiasis in rats to promote stent encrustation is carried out with dietary manipulations, gastrointestinal resections and the administration of lithogenic agents [92]. These animals are often chosen for the validation of both urethral and ureteral stents. Ureteral stents are inserted through a cystotomy in either the bladder or the ureter [106,110,111]. In addition to the evaluation of the device’s performance, when placed in the ureter, uretero-ureteral anastomosis may also be performed for the later histological analysis of ureteral healing and scarring processes [66,110,111]. Urethral stents are tested in the bladder and the urethra, and depending on stent size and characteristics, transurethral placement may be feasible [112,113,114]. The rat’s urethra allows the detection as well as the histological analysis of injuries during stent placement and the development of urethral strictures secondary to fibrotic and hyperplastic tissue formation [114].

##### Rabbits

The rabbit has been used for biocompatibility studies of stent materials. To this end, stent samples can be inserted in the muscle by blunt dissection, preferably the dorsal muscles to prevent the animal from self-mutilation [115]. The scientific literature regarding urinary stent validation in this animal model is scarce, which is probably due to the significant differences between rabbit’s and human’s urine composition [116]. The potential of biomaterials and drug-release against stent-related urinary tract infections has been assessed by transurethral intravesical placement of ureteral stent samples for microbiological cultures and histological analysis [117,118]. The rabbit’s urethra enables the evaluation of urethral and prostatic stents including placement, degradation of materials, therapeutic success and histology in both healthy and urethral stricture models [119,120].

## 6. Concluding Comments

Before implementing a urinary stent modification or innovation into clinical practice, the necessary steps to be taken are in silico, in vitro, ex vivo, in vivo and human trials. Three crucial steps between the computer’s drawing board (in silico simulation) and insertion into human bodies must be taken, namely in vitro, ex vivo and in vivo testing.

All models are suffering from difficulties to accurately reflect conditions in a highly variable and complex environment that is the urinary system.

In vitro models are mostly used to assess the encrustation, durability, and bacterial resistance of stents. Whereas they can give valuable insights into the underlying mechanisms of biofilm formation and encrustation, they remain far from reflecting true conditions within the urinary tract. For the future, examination of the urinary microbiome may provide promising insight into the underlying mechanisms of biofilm formation and encrustation on urinary stents. It has been suggested that the urinary tract is not, contrary to earlier assumptions, a perfectly sterile environment, and that commensal bacteria may play a role in patient susceptibility to infection and in the composition of the urinary microbiome associated with stent complications [121]. Therefore, OMICs (genomics, transcriptomics, proteomics and metabolomics) have improved our understanding of microbial interactions in the urinary tract. It is now possible to identify all microbial species that colonize the urinary tract. Combining results from OMICs studies with in vitro biofilm research has the potential of making a real impact in clinical practice in the future.

The next step would be ex vivo experiments. The necessary considerations to set up a successful experiment with an animal organ on the laboratory bench have been discussed above, taking a biodegradable stent as an example. Definition of the success endpoints related to ex vivo research so that it can be successfully translated to in vivo work is needed. These criteria should include but are not limited to the following stent-related endpoints [10,13]:

- The biocompatibility of the polymers used is a determining factor in the proper function of the device.
- Their chemical structure must contain labile groups that facilitate their cleavage.
- The choice of degradation time will be made according to the indwelling time of a conventional stent in clinical practice, for each specific indication.
- Biodegradable devices may obstruct the urinary tract with degradation fragments, which represents one of the main obstacles to research in this area.
- As for the antifouling performance of stents, ex vivo experiments are not decisive. In vivo and ex vivo experiments need to be compared.
- The laboratory tests must be performed correctly so that the results are compatible with the human organism.

Finally, for reporting animal research, it is recommended to follow the ARRIVE guidelines. These guidelines have been developed to ensure that studies involving live animals follow methodological rigor, are reported in enough detail, and enable reproducibility. This tool is primarily aimed for the writing and revision of scientific publications. However, they are also valuable for study planning and conducting, as they help researchers to design rigorous and reliable in vivo experiments, minimize bias, and to record important information about study methods. In addition, ethical review boards, funders, institutions and learned societies may rely on them to help promote best practice and ensure the rigorous design and transparent reporting of in vivo preclinical research [122].

ENIUS brought together all parties interested in urinary stent improvements. Throughout the project, it became clear that many researchers are working on very limited aspects of stent research, being unaware of the bigger picture, namely the final implementation of a research product into clinical practice for the benefit of patients. Therefore, a collaboration such as ENIUS is not only laudable but also mandatory to combine each disciplines’ strengths and amend their weaknesses. We hope this paper can act as a stepping stone toward that goal.

## Figures and Tables

**Table 1:** Selected in vitro models for the investigation of encrustations and biofilms on urological devices.

Method	Description	Advantage	Limitation	References
**flow models**	**open system** fed-batch process continuous process			
**CFSTR**	identical conditions in the reactor	control of media, flow, biofilm growth	no natural urine flow (peristalsis)	
CFSTR	dynamic flow encrustation model	first publication of an in vitro model to simulate the urinary tract	relatively low throughput	Finlayson and Dubois, 1973 [15]
CFSTR	dynamic flow encrustation model, artificial urine	long-term testing of materials	urease-induced precipitation	Cox et al., 1987 [35]
CFSTR	variation of Finlayson chamber	sterile and infected urine	only for material samples	Gleeson et al., 1989 [36] Stickler et al., 1999 [37]
CFSTR	validated encrustation model	in vitro results correlated with in vivo studies	relatively low throughput	Choong et al., 2000 [38]
CFSTR	encrustation model	a large number of biomaterials in replicates and over long periods of time can be assessed	urease-induced precipitation, high flow rate (10 mL/min), no peristalsis	Gorman et al., 2003 [19]
CFSTR	CDC biofilm reactor, encrustation model	rapid screening of encrustation, reproducible	only for material samples	Gilmore et al., 2010 [21]
CFSTR	encrustation model reaction vessel with dynamic flow, artificial urine and bacterial cultures.	test chamber under static and dynamic conditions	simple model. results comparable to other models but not significantly excelling	El-Azizi et al., 2015 [39]
CFSTR	reaction vessel with dynamic flow and artificial urine	long-term testing of urinary catheter materials	urease-induced precipitation	Cauda et al., 2017 [40]
CFSTR	dynamic in vitro bladder infection model	test antibiotics with human urine	72 h test	Abbott et al., 2018 [41]
CFSTR	reaction vessel with dynamic flow and artificial urine and bacterial cultures	long-term testing of urinary catheter materials	urease induced precipitation	Frant et al., 2018 [42]
CFSTR	reaction vessel with dynamic flow and artificial urine and bacterial cultures	long-term testing of urinary catheter materials	urease-induced precipitation	Hopps et al., 2018 [43] Goeres et al., 2005 [44]
CFSTR	ureter stent model deformation in ureter-stent configurations	investigation of stent failure in extrinsic ureteral obstruction	physiological urodynamics not included	Shilo et al., 2021 [45]
CFSTR	dynamic in vitro bladder infection model	long-term testing of urinary catheter materials	test on small material samples	Zang et al., 2020 [46] Wang 2019 [47]
**PFR**		control of flow and composition of medium (plugs)		
PFR/MRD	modified Robbins device monitoring of encrustation formation	study of calcium oxalate encrustation in artificial urine	test on discs of 10 mm diameter	Malpass et al., 2002 [48]
PFR/MRD	monitoring of encrustation formation	simulate the upper urinary tract, long-term testing of urinary catheter, artificial urine	relatively low-throughput, urease-induced precipitation	Tunney et al., 1997 [49] Gorman et al., 2003 [19] Laube et al., 2016 [20]
FMC	flow model of crystallization of urinary stone	crystallization of calcium oxalate	homogeneous crystals	Achilles et al., 1995 [50] Bouropoulos et al., 1996 [29] Ananth et al., 2002 [31]
**Static models**	closed system batch process	for assessment of biomaterials in the bladder		
Reaction vessel	model to simulate the urinary bladder	pooled human urine	low encrustation in human urine	Getliffe et al., 1994 [51]
Reaction vessel	reaction vessel in an incubator	crushed kidney stones in artificial urine and in human urine	relatively low throughput	Tunney et al., 1996 [52]
Reaction vessel	model to simulate the urinary bladder	cultured daily to determine bacterial growth	relatively low-throughput	Gaonkar et al., 2003 [53]
Reaction vessel	reaction vessel on heating plate	artificial urine	urease induced precipitation	Jones et al., 2006 [54]
**MTP**	biofilm formation assay	opportunity for multiplexing and screening	test on small material samples	Silva et al., 2010 [24] Wilks et al., 2021 [33]
current technologies				
**SoC**	stent-on-chip microfluidic model	examination of the stent design, encrustation and malfunctions	physiological urodynamics not yet included	Mosayyebi et al., 2019 [32]

Legend: CFSTR = Continuous Flow Stirred Tank Reactor; PFR = Plug Flow Reactor; MRD = Modified Robbins Device; MTP = Microtiter plate; CDC = Center for Disease Control; FMC = Flow model of crystallization; SoC = Stent-on-Chip-Model. (Bold font and grey background mark headers and sub-headers).

## Data Availability

The data presented in this study are available on request from the corresponding author.
